# On the Mal d'Aleppo

**Published:** 1814-04

**Authors:** John Girdlestone

**Affiliations:** Yarmouth


*March* 5, 1814.

---

*For the Medical and Physical Journal.*

*On the Mal d'Aleppo; by* Dr. JOHN GIRDLESTONE.

IN the ingenious work of Dr. Bateman on the Diseases of the Skin, I do not perceive any notice taken of the Mal d'Aleppo. Above thirty years ago, I saw a case of the *male Mal,* in the Tanjore district of India. And soon after the 2d edition of Dr. Alexander Russell's History of Aleppo, by his brother Dr. Patrick Russell, appeared in 1794, I had a case of the *third species of Mal,* in this place, in a female, who, I may venture to say, had never resided out of this her native country. The eruptions were about six or seven in number, situated on the forehead and cheek of the same side. I profited by the advice of Dr. Russell in leaving to nature this disease, which, within the course of nine or ten months, gradually disappeared, without leaving any serious deformity of the skin. As the disease is curious, and the book too expensive for many of your readers, I should recommend your citing from this splendid work the whole description.*

JOHN GIRDLESTONE, M.D.

*Yarmouth, March* 5, 1814.

*For*

---

* The Editors are obliged to Dr. Girdlestone for his suggestion. They have copied the account of the disease from the first edition of Dr. Russell's valuable work, which happened to be in their possession. Should Dr. G. however, find any material alteration in the later edition, they will be glad if he will point it out.

" *Mal d'Aleppo.*—A cutaneous disease, thought by some to be peculiar to this place, has acquired the name of *Il mal d'Aleppo,* or *Aleppo evil,* among the Europeans. The natives call it *Habt il senne,* or *Botch of a year,* from the supposed time of its duration. In Turkish, *Haleb Choban,* or *the Aleppo ulcer.* This disease is not, however, peculiar to this place, being almost as common at Antab, and all the other villages on the banks of the rivers Sejour and Coick, as at this place; which favors the opinion of its being occasioned by the water.

" The natives reckon but two species of this disorder, and distinguish them by the names of male and female; but there is a third kind of cutaneous distemper, which, though it is commonly ascribed to to the bite or sting of a common millepedes, or wood-louse, seems to me to be altogether of the same nature, though milder in degree.

"What they call the male distemper, makes its appearance in the form of a small, red, hard tubercle or pimple, which commonly passes some weeks unregarded, as it gives no manner of uneasiness: afterwards it begins to increase, and usually comes to the size of an English sixpence, which, after some months, begins to be scurfy on the top; by degrees the little matter that oozes out of it, forms into a thick crusty scab; which, unless it is picked off, or otherwise disturbed, remains upon it until the parts underneath being healed, it falls off, and leaves but a very small mark. The whole of its duration is seldom above eight months.

"What is called the female species begins like the former; but after a month or two it becomes somewhat painful, increases often to double the extent of the male, discharges a good deal of the ichorous matter from under the scab, and by degrees comes to have the appearance of an indigested scorbutic ulcer, with a livid circle round it, but seems to be no deeper than the *tunica cellulosa.* In this condition it remains for several months, and is in general about a year from its first appearance before it is cured; but this is not a thing certain, many getting well some months sooner, while others remain several months longer. After it is cicatrised, it leaves an ugly scar, which remains through life, and for many months has a livid color. When they are not irritated, they seldom give much pain.

"The third kind of *Mal,* which they call the pinch of a millepedes, begins like the two others, but seldom grows larger than about twice the size of a large pin's head, and never changes its appearance, remaining a small tubercle for many months, without any pain, after which it usually throws off a few scurfy scales and disappears; but some remain a much longer time.

"It affects the natives when they are children, and generally appears in the face, though they also have some on their extremities; for most of them have two, three, or sometimes more, it being rare that that they have but one. In strangers, it commonly appears some months after their arrival; and they have them not so frequently on the face as the natives: very few escape having them, but they seldom affect the same person above once; dogs and cats are as subject to the disease as men; it commonly breaks out upon the nose of these creatures.

"In respect to the cure, like the tooth-ach or ague with us, every one pretends to an infallible remedy for them; but the many beautiful faces daily impaired by the disease, are too evident proofs of their ill success: and in truth, from what I have observed, it is infinitely better to apply nothing, than any of the numberless medicines they make use of.

"Of several applications that I made trial of upon myself and some others, I found the mercurial plaister the most efficacious; the prescription was the same as the *Emplastrum commune cum mercurio*, with a smaller proportion of mercury, and a little larger of *Bals. sulphur.*

"If this was applied at the beginning, it often prevented their making any further progress; if they had begun to run, it hindered them from increasing so much as they would otherwise have done, and generally cured them before their usual time. This is to be understood of that called the female; for the male, as well as the third kind, seldom require any medicinal application."

